# Robot-assisted voluntary initiation reduces control-related difficulties of initiating joint movement: A phenomenal questionnaire study on shaping and compensation of forward gait

**DOI:** 10.1371/journal.pone.0194214

**Published:** 2018-03-12

**Authors:** Patrick Grüneberg, Hideki Kadone, Naomi Kuramoto, Tomoyuki Ueno, Yasushi Hada, Masashi Yamazaki, Yoshiyuki Sankai, Kenji Suzuki

**Affiliations:** 1 Institute of Liberal Arts and Sciences, Faculty of Global Standard Education, Kanazawa University, Kanazawa, Japan; 2 Center for Innovative Medicine and Engineering, University of Tsukuba, Tsukuba, Japan; 3 Graduate School of Empowerment Informatics, University of Tsukuba, Tsukuba, Japan; 4 Department of Rehabilitation Medicine, University of Tsukuba Hospital, Tsukuba, Japan; 5 Department of Orthopaedic Surgery, University of Tsukuba, Tsukuba, Japan; 6 Center for Cybernics Research, University of Tsukuba, Tsukuba, Japan; University of Illinois at Urbana-Champaign, UNITED STATES

## Abstract

Humans employ various control strategies to initiate and maintain bodily movement. In case that the normal gait function is impaired, exoskeleton robots provide motor assistance during therapy. While the robotic control system builds on kinematic gait functions, the patient’s voluntary efforts to initiate motion also contribute to the effectiveness of the therapy process. However, it is currently not well understood how voluntary initiation as a subjective capacity affects the physiological level of motor control. In order to understand the functional nexus between voluntary initiation and motor control, we interviewed patients undergoing robotic gait rehabilitation with the HAL exoskeleton robot about their experience and command of voluntarily initiating forward gait while using the HAL system. Their reports provide phenomenal evidence for voluntary initiation as a distinct cognitive act that comes as phenomenal performance. Furthermore, phenomenal evidence about the functional relation of intention and initiation correlates with FIM-M gait scores. Based on the assumption that HAL reduces control-related difficulties of voluntarily initiating joint movement, we identified two cognitive control strategies, shaping and compensation of gait, that imply a heterarchic organization of the human system of action control.

## Introduction

The purpose of robotic systems for therapy of upper [1] and lower limbs [2] is to support patients in executing motions that they cannot anymore execute by themselves. Recent advances in this new field of robot-assisted therapy suggest that the active participation of the patient in the therapeutic process has positive effects on motor recovery [3]. I.e. the patient’s voluntary involvement while using a robotic device supports neuroplasticity for therapeutic purposes and motor learning. However, voluntary resources are not considered in the standard electromechanical approach to robot-assisted therapy. Most exoskeleton robots (as well as traditional physiotherapy) act on the (distal) physical level in order to influence the neural system. This approach leaves the patient passive: the impaired limbs of a patient are being moved.

**Fig 1:**
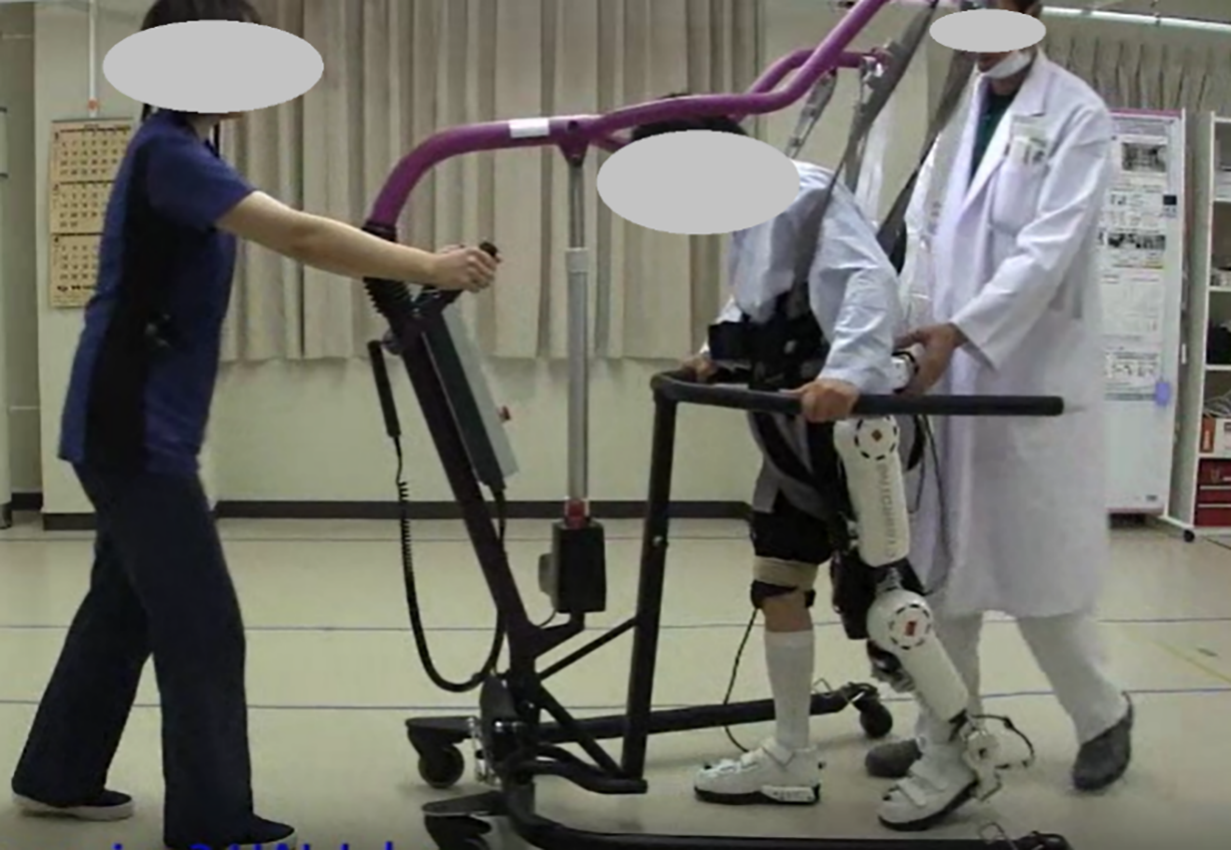
HAL patient during therapy.

In order to fully exploit the active participation of the patient, an alternative control strategy builds on biosignals [4] within the proprioceptive loop of movement initiation, execution and feedback to the brain. Even in the case of severely impaired patients, efferent EMG signals of neural muscle activity often remain in the muscles, but do not anymore result in sufficient joint movement. The exoskeleton robot HAL (hybrid assistive limbs; Fig 1) detects and interprets these signals [5] as the patient’s intention to move his body voluntarily [6]. Accordingly, the patient activates the robot by means of voluntary initiation [VI] of forward gait [7], instead of being moved passively by predetermined kinematic patterns. Preliminary studies of HAL-assisted therapy provide provisional clinical data regarding beneficial effects on gait therapy [8] ranging from stroke patients [9][10][11][12][13] to patients with spinal cord infarction, musculoskeletal and other diseases [14][15][16] and one patient with ossification of the posterior longitudinal ligament (OPLL) [17].

Currently, HAL works with a broad variety of impairments. Regarding the interaction between patient and robot, any EMG-signals in the leg muscles–regardless the respective diagnosis–are interpreted in a standardized manner as the patient’s intention to move. Despite the implementation of HAL, it is still unknown how VI actually operates on motor programs. In the face of increasing evidence that volitional capabilities play an efficacious role in movement control, the importance of investigating subjective execution of volitional acts has been stressed recently [18][19]. Whereas such volitional capabilities are usually ascribed to a human subject of movement [20], current research in movement and its behavioral underpinnings is mostly concerned with kinematic aspects [21]. In turn, philosophical accounts of volition [22] do not focus on the physical implementation of volitional acts. Phenomenological psychology of the early 20^th^ century investigated VI as a distinct phenomenal act [23][24][25][26], but vanished in the advent of behaviorism. Also current phenomenological and cognitive (neuro)science research on bodily movement focuses merely on different types of *sense of* agency and ownership [27][28][29]. Yet, any sense of agency does not cover the genuine act of initiating a movement that is left to subpersonal processes in the brain and not to the conscious subject. In sum, currently hardly any account or data *simultaneously* considers the subjective dimension of VI as an efficacious capacity and its physical implementation in joint movement. The functional relation between volitional acts and bodily movement is rather omitted.

In order to understand how VI operates on motor programs, it is necessary to investigate the subjective (first-person) perspective of the human user during VI of bodily movement. Opposed to healthy subjects who usually do not have a particular consciousness of movement initiation [30], HAL patients have experiences of *“being able to move”* (before their impairment), *“not being able to move”* (due to their impairment) and *“again (partially) being able to move”* (in the course and after therapy). Based on these diverging experiences, they are qualified to identify VI as a particular cognitive act and specify its role in the system of action control. Whereas introspective accounts were disregarded in the sense that introspectively described states or acts do not exhibit any functional (causal) relevance [31], the HAL setting renders these reports relevant in that these describe efficacious phenomenal behavior [7]. Based on these reports and the functional role of VI in the proprioceptive loop of initiation, execution and feedback of bodily movement, we aim to test a set of two hypotheses:

- H_1_: *VI comes as a phenomenal performance*.
- H_2_: *VI is a cognitively distinct act of action control*.

For this purpose, we interviewed HAL patients about their first-person execution of VI while controlling the HAL robot during therapy sessions. We then combined the phenomenal (subjective) evidence for VI with objectively measured gait scores. Hereby, we introduced a novel method for modelling subjective acts (VI) in relation to their physical implementation in joint movement showing that the subjective (voluntary) conditions of the patients correlates with their physiological motor functions. With this method, it was possible to confirm VI as a conscious and cognitively distinct act as well as to specify two cognitive control strategies that patients employ in order to initiate forward gait. These strategies imply two respective modes of robot-assisted VI, revealing that HAL reduces control-related difficulties of voluntarily initiating joint movement.

## Results

**Fig 2:**
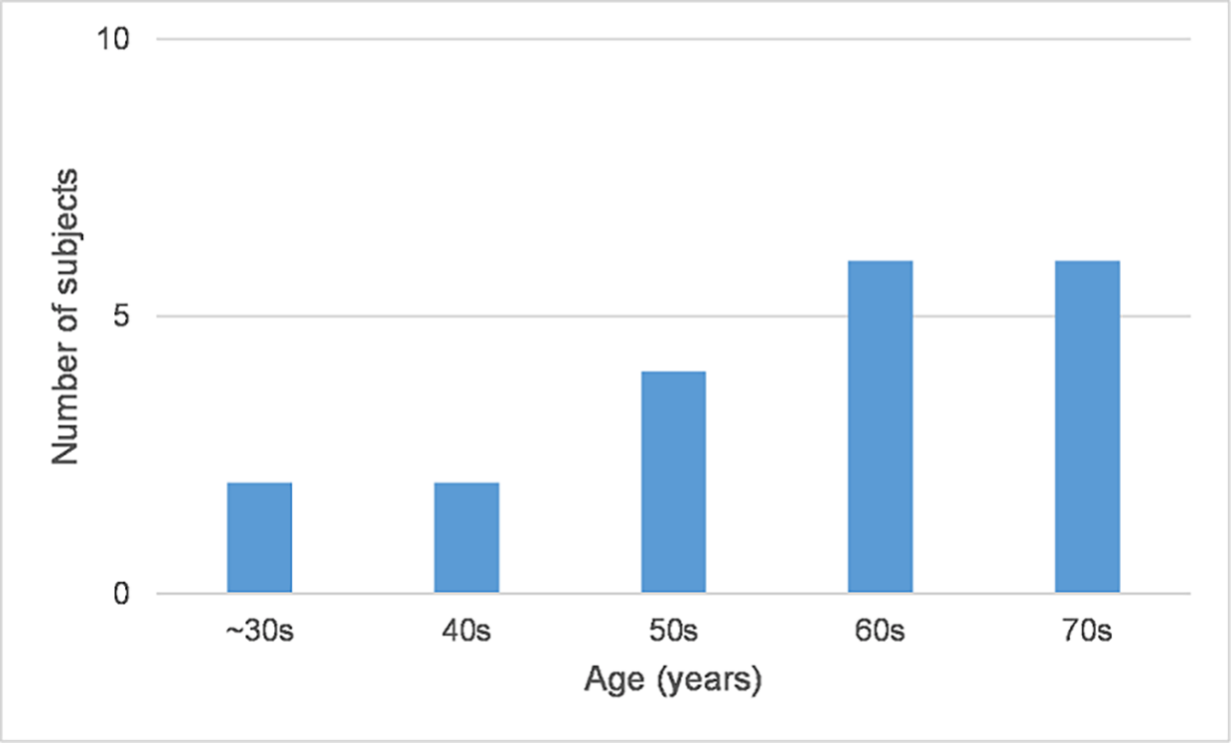
Age and gender of subjects. 12 male, 8 female subjects; n = 20, mean age 59.1, SD ± 16.58 years.

**Fig 3:**
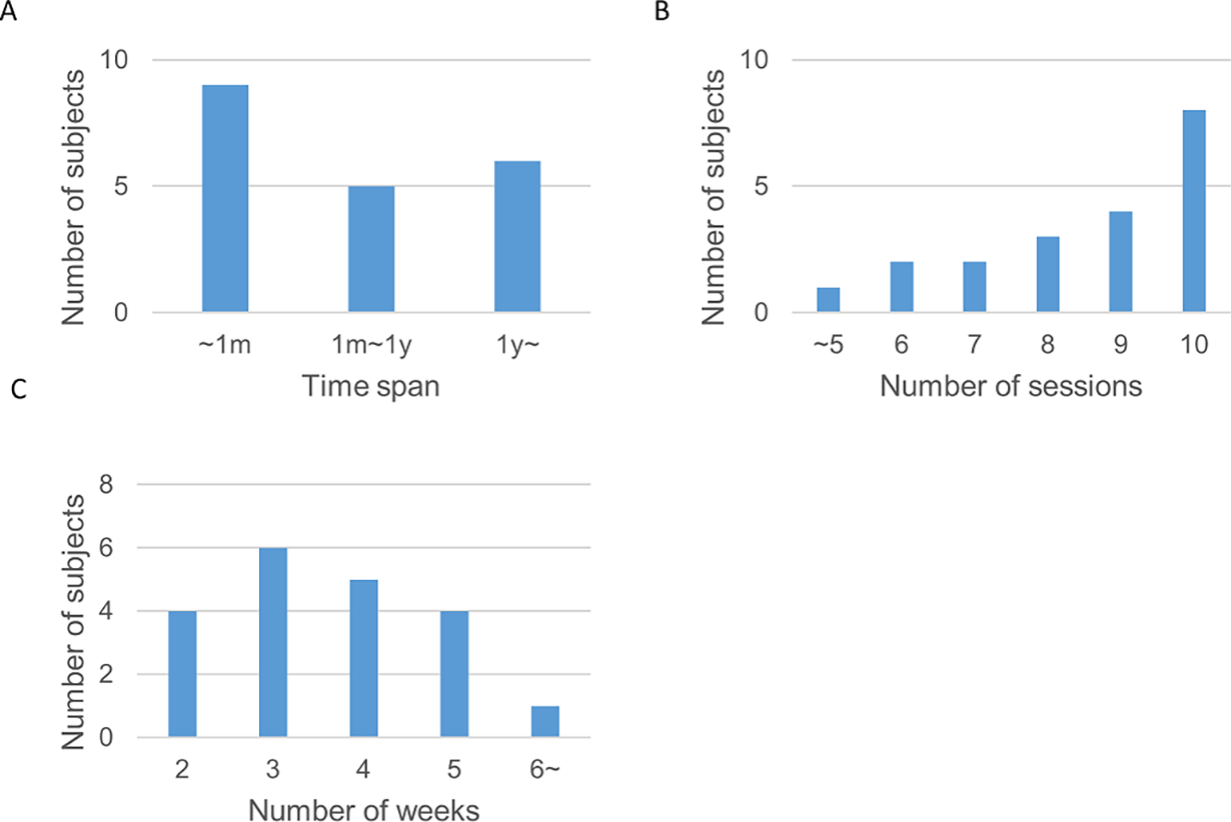
Histogram of participation in therapy. (A) Time span between diagnosis and start of HAL sessions (n = 20). (B) Total number of HAL sessions (n = 20, mean 8.45, SD ±1.87). (C) Number of weeks of HAL sessions (n = 20, mean 3.8, SD ±1.79).

We collected data by means of interviewing 20 HAL patients at University of Tsukuba Hospital. After the patients completed their HAL therapy program, we used a multiple-choice questionnaire with an additional option for comments to every question in order to investigate the first-person (phenomenal) experience while using the HAL robot. The questionnaire [32] consists of totally 25 questions, grouped in four items. In a second step, the results of the questionnaire were correlated to gait scores pre and post therapy (see Materials and methods for more details). The subjects’ age range from one young patient (12 years) to one 79 year-old patient with average age of 59.1 years (**±** 16.58; Fig 2). The conditions of the subjects range from stroke induced diagnoses to spinal cord infarction and neurological diseases (Table 1). All diagnoses impaired the gait function according to the International Classification of Functioning, Disability and Health (ICF b760 Control of voluntary movement functions; b770 Gait pattern functions; d450 Walking). Subjects joined the therapy program during acute and chronic stages (time span between diagnosis and start of HAL sessions lies between less than one month and more than one year; Fig 3A) whereby this condition did not affect the results. The rehabilitation program included average 8.45 (±1.87) HAL sessions per patient (Fig 3B) and average 3.8 (±1.79) weeks of HAL sessions per patient (Fig 3C). During the interview, subjects did not always answer to all questions. In some cases, qualitative answers provided additional information. Due to the explorative nature of this study, not all items provided results relevant for the hypotheses (for an overview of all results see [33]).

**Table 1:** Diagnoses of subjects.

Diagnosis	Total	Male	Female
**Cerebral disorder**	11	5	6
**Spinal disorder**	9	7	2

### Voluntary initiation as phenomenal performance

All subjects confirm that they intentionally participated in the HAL therapy (item 1.1: p < .00001; Table 2), that they were willing to walk forward (item 1.2: p < .00001; Table 2) and that they initiated forward walking by themselves (item 1.3: p < .00001; Table 2). Thus, from a phenomenal first-person perspective, subjects are fully aware of VI. Regarding the phenomenal quality, VI involves motor imagery [34] (item 2.1: p = .00002; Table 3). More specifically, subjects report about dynamic mental imagery, i.e. they have an internal image of the sequence of their walking motion while executing the movement [35] (item 2.2: p = .0033; Table 3). Regarding the phenomenal type, 10 subjects characterized VI as a phenomenal performance (item 2.4: p = .00008; Table 3); 7 subjects rely on thought (p = .00967) that occurs in two cases simultaneously with the performance. E.g., subject 11 explains that she thinks and judges whether her motion is appropriate while initiating movement. Taken together, items 1.1–3, 2.1/2/4 suggest phenomenal evidence for H_1_. Other phenomenal types of action-related consciousness did not prove to be significant, such as one case of decision-making (p = .16894) that co-occurred with thought and one case of memory (p = .16894).

**Table 2:** Results of item group 1.

1. Subjective experience of voluntary initiation		
	Yes	No
1.1 Intention (n = 20)	20 (p < .00001)	0
1.2 Volition (n = 20)	20 (p < .00001)	0
1.3 Initiation (n = 20)	20 (p < .00001)	0

The columns show the absolute number of answers; statistical significance (two-tail p-value) for all items has been calculated using a binomial test with α = .05.

**Table 3:** Results of item group 2.

2. Phenomenal quality of voluntary initiation				
2.1 Imagery (n = 20)	Yes	No		
	19 (p = .00002)	1 (p = .00002)		
2.2 Type of imagery (n = 19)	Static	Dynamic		
	0	17 (p = .0033)		
2.4 Experiential quality (n = 19) (multiple answers)	Performance	Thought	Decision-making	Memory
	10 (p = .00008)	7 (p = .00967)	1 (p = .16894)	1 (p = .16894)

The columns show the absolute number of answers; statistical significance (two-tail p-value) for all items has been calculated using a binomial test with α = .05.

### Voluntary initiation as a distinct act in the system of action control

Subjects distinguished VI from preceding (intention and urge) and subsequent (joint movement, emotion and motivation) functions of action control. Regarding preceding functions, the identification of VI as a distinct act occurs regardless whether the ability to initiate movement voluntarily depends on the preceding intention [36][37] to join rehabilitation (item 3.1: p = .00739; Table 4) or whether a subject can initiate movement independently of her intention to join therapy (p = .00739). The same applies for the relation between a preceding urge [38] and VI. Subjective evidence strongly suggests an inner urge to walk forward after subjects have been prepared with HAL and are ready to move (item 3.4: p = .00018; Table 4). On the other hand, VI does not necessarily follow from the urge because subjects state that they can do nothing about the urge and just have to walk forward (item 3.5: p = .09442; Table 4) as well as that they initiate forward walking independently of the urge (p = .09442). Thus, VI maintains a distinct functional role during action control because the preceding intention and the occurrence of an urge do not necessarily imply the execution of joint movement.

**Table 4:** Results of item group 3.

3. Functional embedding in action control				
Intention				
3.1 Relation of initiation to intention (n = 19)	Dependency		Independency	
	4 (p = .00739)		15 (p = .00739)	
3.2 Body movement during rehabilitation (n = 20)	As desired		Not as desired	
	12 (p = .12013)		8 (p = .12013)	
3.3 Relation of intended movement and initiation (n = 13)	Body does not move as intended		Body moves as intended	
	Can initiate	Cannot initiate	Can initiate	Cannot initiate
	10 (p = .00011)	2 (p = .2059)	1(p = .10295)	
Urge				
3.4 Occurrence of urge (n = 20)	Yes	No		
	18 (p = .00018)	2 (p = .00018)		
3.5 Relation of urge to initiation (n = 17)	Automatic	Independent		
	11 (p = .09442)	6 (p = .09442)		
Motivational system				
3.6 Effects on emotion (n = 20)	Positive	Negative	Positive and negative	Neither
	16 (p < .00001)	0	2 (p = .06695)	2 (p = .06695)
3.7 Effects on motivation (n = 20)	Increase	Decrease	Increase and decrease	No effect
	16 (p < .00001)	0	1 (p = .02114)	3 (p = .1339)
3.8 Rehabilitation and motivation (n = 19)	Positive	Not positive	Either or	No effect
	16 (p < .00001)	0	2 (p = .08034)	1 (p = .02678)
3.9 Change of motivation (n = 16)	Increase	Decrease		
	16 (p = .00002)	0		

The columns show the absolute number of answers; statistical significance (two-tail p-value) for all items has been calculated using a binomial test with α = .05.

Regarding subsequent functions, subjects distinguish between VI and the implementation of bodily movement. For the execution of VI, it seems to be irrelevant whether the body moves as desired (item 3.2: p = .12013; Table 4) or not as desired (p = .12013). Furthermore, subjective exercise of VI can occur independently of an actual movement (item 3.3: p = .00011; Table 4). Finally, the data suggests that VI affects emotion positively (item 3.6: p < .00001; Table 4) and increases motivation for therapy [39] (item 3.7: p < .00001; Table 4). In turn, the data does not suggest that there is no effect on emotion (item 3.6: p = .06695) and motivation (item 3.7: p = .1339). Subjects confirm a positive impact of successful movement on motivation (item 3.8: p < .00001; Table 4) and the corresponding increase of motivation (item 3.9: p = .00002; Table 4). In sum, subjective evidence indicates VI as a cognitively distinct act in the system of action control (H_2_).

### Two groups of robot-assisted voluntary initiation

**Fig 4:**
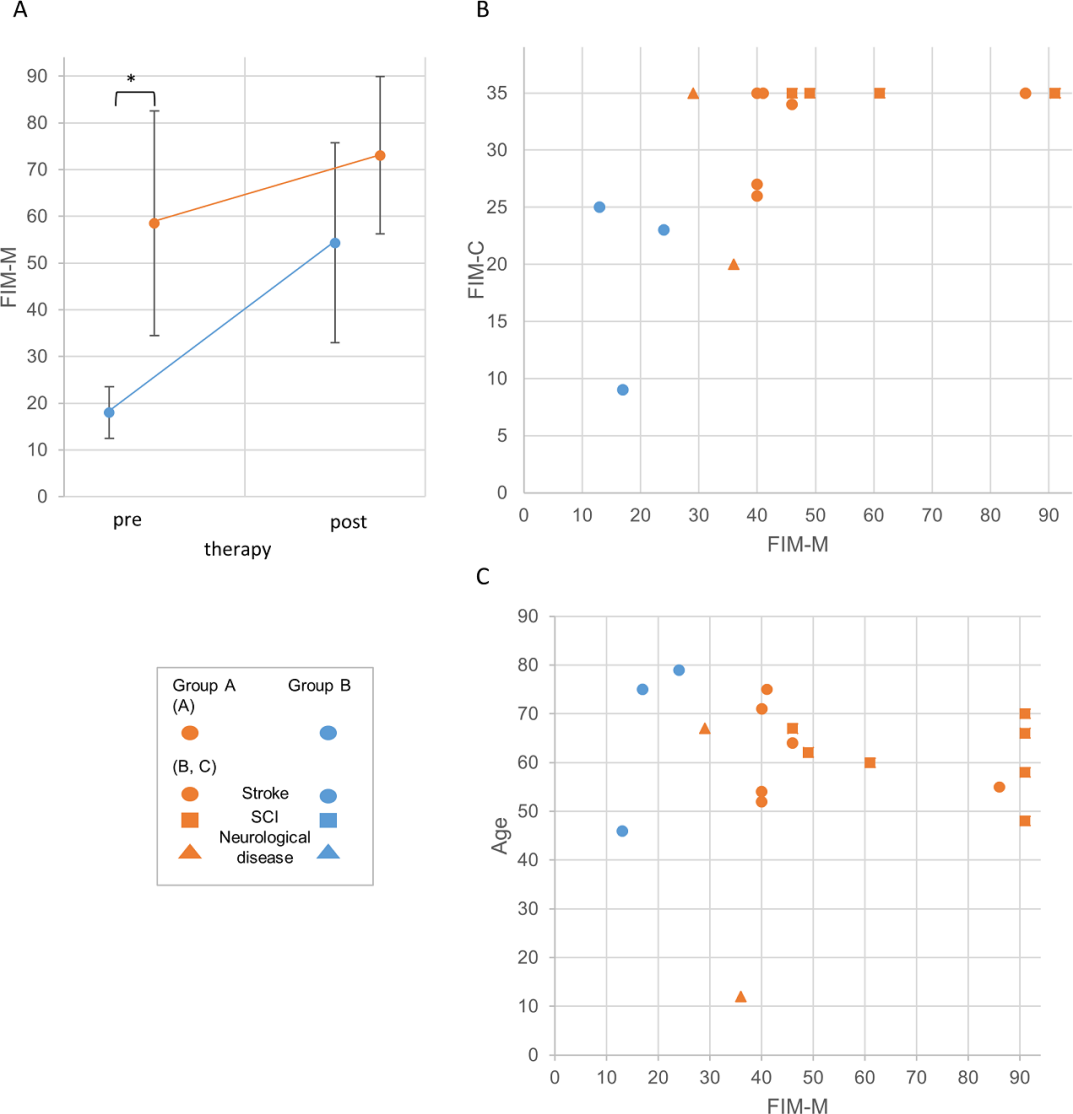
Distribution of FIM-M over two groups of robot-assisted initiation. (A) Distribution of FIM-M scores pre- and post-therapy regarding group A (FIM-M > 24) and group B (FIM-M ≤ 24) (Group A pre: mean 58.533, SD 24.053, post: mean 73.067, SD 16.867; Group B pre: mean 18, SD 5.568; post: mean 54.333, SD 21.385; * n = 20, X^2^ = 10.322, df = 1, p = .00131). (B) Distribution of FIM-M (0–91) and FIM-C (0–35) scores over group A and B. (C) Distribution of FIM-M (0–91) and age over group A and B.

Based on H_1_ and H_2_, a matching of phenomenal evidence for VI with the gait scores of the subjects derived a correlation between the relation of VI to intention (item 3.1) and the FIM-M pre-therapy gait score. Results imply two groups (X^2^ = 10.322, df = 1, p = .00131; Table 5): Members of group A show a pre-therapy FIM-M > 24 and explain that they can voluntarily initiate forward walking *independently* of the preceding intention to join therapy (item 3.1: p = .00739; Table 4). Members of group B show a pre-therapy FIM-M ≤ 24 and explain that their ability to initiate forward walking *depends* on their intention to join therapy (item 3.1: p = .00739). No member of group B has a higher score of FIM-M pre therapy than any member of group A. According to our data, FIM-M ≤ 24 seems to imply a cognitive border below which VI alone is insufficient for initiating voluntary movement (however, the exact value of this border may vary in other data sets). The correlation between VI and FIM-M holds for the condition of the subjects pre-therapy (Fig 4A) and regardless of FIM-C pre-therapy (Fig 4B) or age (Fig 4C).

**Table 5:** Correlation of FIM-M pre therapy with item 3.1 (relation of initiation to intention).

Subject ID^∇^	3.1 Relation of initiation to intention	FIM-M pre therapy
1	Group B: dependency	—
10		13
2		17
11		24
15*	Group A: independency	29
7		36
18		40
17		40
12		40
20		41
14		46
16		46
13		49
9		61
19		86
Chi-Square Test of answers to item 3.1 resulting into group A (FIM-M > 24) and group B (FIM-M ≤ 24): X^2^ = 10.322, df = 1, p = .00131		

∇ Subjects 3, 4, 5, 8 are not considered relevant due to maximal gait scores for FIM-M. For subject 1 only FIM-M/C post-therapy scores exits, for subject 6 no scores exist.

* Subject 15 forms an exception compared to other subjects: effects of rehabilitation are comparably low (FIM-M 29/31, BI 30/35) so that the low FIM-M value is possibly rather due to the subject’s chronic condition than to the capability of VI.

## Discussion

### Subjective evidence for voluntary initiation of joint movement

Results building on introspective reports, particularly in the case of subjects with cognitive impairments, raise questions about the reliability of the responses. With respect to the contents of the responses, the results of this study conform with the acknowledged contribution of motor imagery to the therapeutic process [35][40]. With respect to the method, first, medical doctors recommended all subjects and qualified them as being capable to participate. Second, in most cases FIM-C was rather high. Even in the cases of lower FIM-C, there were no cases of impossible communication or misunderstanding during the interviews. In sum, there seem to be no substantial limitations to the reliability of the responses although a higher number of subjects with a low FIM-M is necessary in order to strengthen the support for the hypotheses.

A further possible bias of the results concerns the comparability of subjects with different disorders.

However, from a clinical viewpoint, the question is (independently of the specific disorder and the disease process) whether a patient is willing to initiate movement or not. Accordingly, the questionnaire focuses on the particular cognitive issue of VI of joint movement and does not aim at evaluating physical performance (FIM-M) or other cognitive issues (FIM-C). Furthermore, diagnoses and disease stage did not affect the responses. Thus, subjects are treated equally only with respect to VI so that comparability within a heterogeneous population could be reasonably assumed.

### Efficacious action consciousness

Results regarding the phenomenal quality and type suggest that VI does not fit in the available phenomenological classification of voluntary acts [41][27][42][43] and implies a genuine type of voluntary action. This subjective evidence confirms the theoretical model of VI [7], according to which VI comes as efficacious action consciousness that bears phenomenal content of the immediate execution (performance) of an action (H_1_). Regarding its individuation (H_2_), VI as the “conscious initiation (the implementation of a decision or intention to act in actual behavior)” can be distinguished from the intention as “practical decision-making (deliberation and termination of deliberation in favor of one possible action)” [44] and other functions related to action control.

In contrast, neuropsychological approaches to action control do not ascribe any efficacious function to volition. According to the WWW model [45], intentional action builds on decisions about which action to execute (*what* component), when to execute an action (*when* component) and whether to execute an action (*whether* component). This classification does not clearly specify VI in the functional terms as suggested by the HAL scenario because the *what* component is part of action planning. Therefore, it comes as what we specified as intention and does not bear any immediate efficacy on joint movement. The *when* component is an essential aspect of VI, but it is not clear in the WWW model whether timing includes the actual execution of movement. The *whether* component is too narrowly defined in terms of intentional inhibition of action. Neither can intentional accounts capture VI as a phenomenal *and* efficacious capacity. Based on attempts to reduce initiation to intention, different classes of intentions are suggested [36][37]. Distal intentions are concerned with the nonimmediate future, proximal intentions are supposed to exhibit their causal role on behavior through intention acquisition, and motor intentions directly work on the physical motor system. However, due to the representational stance, intentions do not bear the functional capacity to operate on motor programs [7][22].

### Heterarchic organization of action control

**Fig 5:**
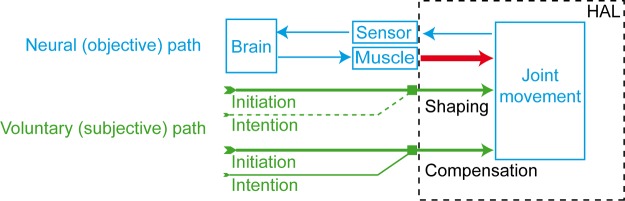
Neural and voluntary pathways of shaping and compensation. The red arrow in the neural path shows the functional location of gait impairment. The dotted line in the voluntary path of shaping (group A) shows that intention is present, but not necessary for initiating joint movement.

The phenomenal and causal characteristics of VI give rise to questions about the functional organization of the system of action control. In particular, the results of item 3.1 are relevant because they correlate with the objective condition of the subjects and thereby describe a relation between VI and the motor level. According to the phenomenal report and the FIM-M pre-therapy value, two cognitive control strategies and two respective modes of robot-assisted initiation can be distinguished based on the three functions of (1) intention, (2) VI and (3) joint movement (Fig 5):

- *Shaping*: Subjects of group A execute VI to the extent that they are able to initiate movement independently of any previous intention. This subjective condition correlates with a FIM-M > 24. Because these subjects control HAL by means of VI, HAL supports the subsequent implementation of joint movement, i.e. *shapes* their gait function.
- *Compensation*: Subjects of group B cannot execute VI as an independent capacity. Instead, HAL enables these subjects to utilize additional intentional efforts in order to initiate joint movement and thereby *compensates* for insufficient VI.

The two modes suggest different configurations of action control in a heterarchic system. Opposed to common hierarchical views of action control where the governing function is predetermined, intention, initiation and joint movement do not stand in a linear, monocausal nexus from the former to the latter. In a heterarchic system, a configuration consists of the reciprocal activity of independent functions. Depending on the situation and needs of the agents, one of these functions governs the overall system [46][47] so that an agent is able to employ intention and initiation simultaneously (compensation) or independently (shaping).

In general, there is hardly any related work on cognitive control strategies of joint movement. The identification of voluntary gait patterns (opposed to automatic gait patterns) during the activation of gait synergies [48][49] suggests preliminary physical evidence for VI as the cognitive origin of these voluntary patterns. Additionally, phenomenological analysis of introspection by Ach can serve to clarify the employment of the two modes [23][50][51]. The “law of difficulty” states that *voluntary effort increases as a positive function of the perceived difficulty of implementing an intended action* [52] and thereby links the subjective perspective with the physical condition of the agent. Accordingly, HAL reduces control-related difficulties: subjects of both groups are enabled to execute VI despite their impairment whereby subjects of group B employ additional intentional efforts. Further investigation has to specify to what extent the impairment of members of group B originates in VI or the motor level respectively. The report of all members of group B about VI as a phenomenal performance (item 2.4) suggests the assumption that they actually execute VI. Hence, the impairment may be rather due to motor defects that disable VI.

### Clinical implications

Knowledge about modes of efficacy of VI could provide more fine-grained criteria for the interpretation of EMG-signals through the robot. For this purpose, it would be necessary to identify variations in gait patterns and related EMG-signals according to the cognitive control strategies. This refinement could deepen the integration of robotic devices in the execution of volitional acts and allow for a more individualized robotic support of specific impairments.

## Materials and methods

### HAL therapy session

HAL (Hybrid Assistive Limb, Cyberdyne, Tsukuba, Japan) is a wearable type assistive exoskeleton robot for the lower limbs. It has segments to be attached to the waist, thigh, shank and foot. The single leg version of the robot was applied for stroke patients, and dual legs version for the others. The robot has electric motors to assist joint motion of hip and knee. The motors are actuated in one of the two modes: CVC (Cybernic Voluntary Control) and CAC (Cybernic Autonomous Control) mode. In CVC mode, the motors are driven according to neuro-muscular activation detected as electric signals measured using electrodes attached on the skin surface corresponding to the muscles for each of flexion and extension of hip and knee joint. By amplifying the detected electric activation as joint torque, CVC mode can support the lower limb motion in real-time according to the motion intention of the user. In CAC mode, the motors are controlled to reproduce a pre-defined trajectory according to foot-floor contact measured by floor reaction pressure sensor in the shoes. CAC mode is used for patients with complete loss of the electric activation on the lower limbs. CVC mode was applied to all of the patients in this study. Parameters for tuning the amplification gain was adjusted manually on site for each patient’s comfort and smoothness of gait.

One session of HAL therapy took 90 minutes, including clinical assessments before using HAL, attaching HAL, walking with HAL, detaching HAL and assessments after the walking training. Time duration of walking with HAL was 10–20 minutes in total in one session. A medical doctor, one or two physical therapists, and an assistant attended each session. For safety reasons, a walking device (All-in-One walking trainer, Ropox A/S, Naestved, Denmark) with a harness protected patients from falls during walking with HAL.

### Experimental procedure

For each of the patients, an interview-based questionnaire was conducted just after finishing the last HAL session [53]. Before conducting the interview, the purpose, method, expected contribution, and privacy protection issues regarding the questionnaire were explained by a medical doctor before written informed consent was obtained. For some of the patients, typically stroke patients who participated in their acute phase, there was a concern that they might have not understood the explanation thoroughly. For these patients, their spouses gave written informed consent. The same experimenter did the interview for all of the patients. The experimenter first asked the patients whether they wanted to read the questionnaire sheet and fill in the answers by themselves. In this case, the experimenter stayed still beside the patients and, in case of any question, supported the patients’ understanding of the questionnaire. Otherwise, the experimenter verbally read out each of the sentences of the questionnaire and filled in the answers based on the patients’ verbal response. Ethical clearance for this research was reviewed and approved by the institutional review board of University of Tsukuba Hospital (Application No. H27-034).

### Interview-based questionnaire

In order to learn about the subjective condition during VI of joint movement, it is necessary to ask subjects about their cognitive behavior in their first-person experience. The relevant method here is *phenomenological introspection*. While there are still substantial doubts about the reliability of introspection [31], it is the only available method for this purpose which has been developed extensively in phenomenological psychology [23][50][51][26]. In contrast to qualitative interview approaches which build on open-ended questions [18], our questionnaire builds on multiple-choice questions (with the opportunity to give remarks at every question) for the sake of quantification [32]. During the interview, item 2.4, concerning the overall phenomenal quality of VI, appears as a summarizing question Q9 after Q8.2 before passing over to item group 4 on control issues.

The questionnaire contains the following items:

1. Subjective experience of voluntary initiation
  1.1 Intention (Q1)
  1.2 Volition (Q2)
  1.3 Initiation (Q3)
2. Phenomenal quality of voluntary initiation
  2.1 Imagery (Q4.1)
  2.2 Type of imagery (Q4.2)
  2.3 Non-pictorial awareness of body parts (Q4.3)
  2.4 Experiential quality (Q9)
3. Functional embedding in action control
  Intention
  3.1 Relation of initiation to intention (Q5.1)
  3.2 Body movement during rehabilitation (Q5.2)
  3.3 Relation of intended movement and initiation (Q5.3)
    Urge
  3.4 Occurrence of urge (Q6.1)
  3.5 Relation of urge to initiation (Q6.2)
    Motivational system
  3.6 Effects on emotion (Q7.1)
  3.7 Effects on motivation (Q7.2)
  3.8 Rehabilitation and motivation (Q8.1)
  3.9 Changes of motivation (Q8.2)
4. Control issues
  4.1 Mental and physical effort (Q10.1, 10.2, 10.3, 10.4, 10.5, 10.6)
  4.2 Futile efforts to move (Q11)
  4.3 Walking speed (Q12)
  4.4 Walking direction (Q13)

### Subjects and statistical data

Twenty subjects participated in the study. The participants were patients at the University of Tsukuba Hospital. All of them had locomotor dysfunction due to damages in the central nervous system. The patients showed different diagnoses for the principal cause of motor impairment. The remaining locomotor function depended on the cause, severity of damage and time from diagnosis. Typically, stroke patients had hemiparesis, and myelopathy patients had paraparesis. Statistical data includes the following:

- Gender: Male / Female
- Age
- Diagnosis
  ○ Cerebral disorder
  ○ Spinal disorder
- Time span between diagnosis and start of HAL sessions
- Total number of HAL sessions
- Number of weeks of HAL sessions

Availability of stroke patients who were capable of participating in the interview was highly limited.

### Clinical assessment

For each patient, clinical assessment was conducted before starting the first HAL session and after finishing the last HAL session. The clinical assessment included evaluation according to FIM (Functional Independence Measure) [54] separately for motor related scores (FIM-M) and cognition related scores (FIM-C), mRS (modified Rankin Scale) [55] and BI (Barthel Index) [56], in addition to a standard 10m or 6m walking test.
